# Allelopathic Activity of Extracts from Different Brazilian Peanut (*Arachis hypogaea* L.) Cultivars on Lettuce* (Lactuca sativa)* and Weed Plants

**DOI:** 10.1155/2017/2796983

**Published:** 2017-03-15

**Authors:** G. S. Casimiro, E. Mansur, G. Pacheco, R. Garcia, I. C. R. Leal, N. K. Simas

**Affiliations:** ^1^Laboratory of Micropropagation and Plant Transformation, Cellular Biology Department, State University of Rio de Janeiro, Rio de Janeiro, RJ, Brazil; ^2^Laboratory of Natural Products and Biological Assays, Department of Natural Products and Food, School of Pharmacy, Federal University of Rio de Janeiro, Rio de Janeiro, RJ, Brazil

## Abstract

Peanut (*Arachis hypogaea *L.) is the fourth most consumed oleaginous plant in the world, producing seeds with high contents of lipids, proteins, vitamins, and carbohydrates. Biological activities of different extracts of this species have already been evaluated by many researchers, including antioxidant, antitumoral, and antibacterial. In this work, the allelopathic activity of extracts from different Brazilian peanut cultivars against lettuce* (Lactuca sativa)* and two weed plants (*Commelina benghalensis* and* Ipomoea nil*) was studied. Aerial parts, roots, seeds, and seed coats were used for the preparation of crude extracts. Seed extract partitioning was performed with* n*-hexane, dichloromethane, ethyl acetate,* n*-butanol, and aqueous residue. Germination and growth of hypocotyls and rootlets were evaluated after one and five days of incubation with plant extracts, respectively. Crude seed extract and its dichloromethanic partition displayed highest allelopathic activity. These results contribute for the study of new potential natural herbicides.

## 1. Introduction

Peanut (*Arachis hypogaea *L., Leguminosae) is the fourth most planted and consumed oilseed worldwide and also a good option for crop rotation. In addition to its flavour, peanut seeds have a high content of lipids (about 50%), proteins (22% to 30%), mineral salts, vitamins, and carbohydrates, being highly energetic, with an average of 585 calories per 100 grams of the grain [1].

The evaluation of different biological activities within the genus* Arachis* led to the recognition of antioxidant, antibacterial, antitumoral, and anti-inflammatory potentials, among others. However, allelopathic activity is comparatively less investigated. Allelopathy is any positive or negative effect of one plant on another through the production of chemicals that are released into the environment [2]. A great importance of plants with allelopathic activity is to fight against weed plants, which are defined as plants that grow where they are not desired, along with agricultural crops, interfering in their development and reducing productivity [3]. Although routinely weed plants are combated with herbicides, their indiscriminate use leads to the development of resistant plants. In addition, the impact of herbicides on human health is also another debatable fact. In this context, the search for natural products instead of synthetic herbicides for weed plant management is highly recommended [4, 5].

To our knowledge, the allelopathic potential has already been studied only in two species of genus* Arachis*. The inhibitory activity of seed extracts in the germination of millet and the effect of root and leaf extracts of* A*.* hypogaea *on rice, maize, radish, and rye grass, besides a positive stimulus of aqueous extracts of* A*.* pintoi* on the germination of tomato seeds* (Solanum lycopersicum)* and pepper* (Capsicum annum)*, have been studied [6–8].

In view of this fact, this paper evaluates the allelopathic activity of extracts from different organs of five Brazilian peanut (*Arachis hypogaea *L.) cultivars against lettuce* (Lactuca sativa)* and weed plants (*Commelina benghalensis* and* Ipomoea nil*).

## 2. Material and Methods 

### 2.1. Plant Material

Aerial parts, roots, seeds, and seed coats of five Brazilian peanut cultivars, named IAC 886 (Virginia Runner), IAC Caiapó (Virginia Runner), IAC Tatu ST (Valencia), IAC 8112 (Spanish), and IAC BR-1 (Valencia), were used in this study. Plants were grown from seeds in the greenhouse of Rio de Janeiro State University, Brazil, in pots containing Plantmax®. Light intensity in a clear day during the growing period was as high as 1600 *μ*E/(m^2^/s). Aerial parts and roots were taken for extraction 30 days after planting.

### 2.2. Preparation of Extracts

#### 2.2.1. Crude Extracts

Plant materials were dried in an oven at 50°C, crushed, and weighed. Soaking in ethanol 100% was preceded by a degreasing step with* n*-hexane (five stages during ten days). Maceration in ethanol was performed in the dark at room temperature (five stages during ten days). After filtration on filter paper and concentration in a vacuum rotary evaporator at 45°C, extracts were weighed and stored at 4°C until used.

#### 2.2.2. Partitioned Extracts

The extract of cultivar BR-1 seeds was resuspended in methanol : distilled water (9 : 1) and subjected to successive liquid-liquid partitioning using organic solvents of increasing polarity, namely,* n*-hexane, dichloromethane, ethyl acetate, and* n*-butanol, followed by concentration in a vacuum rotary evaporator at 45°C.

### 2.3. Evaluation of Allelopathic Activity

#### 2.3.1. Germination of Seeds

Samples of the extracts were dissolved in chloroform and the volume adjusted to the desired concentrations (1000, 500, 200, and 100 ppm). Each petri dish (*d* = 6 cm, *h* = 1 cm) containing filter paper discs Whatman® No. 1 (*d* = 5.7 cm) received 0.5 mL of the extract. After solvent evaporation at room temperature (24 hours), ten seeds (*L*.* sativa*,* C*.* benghalensis,* or* I*.* nil*) and 2.5 mL of distilled water with DMSO 0.1% were added to the petri dish. All assays were conducted in triplicate, with three replications. Menadione in the concentration of 200 ppm served as positive control, while distilled water with DMSO 0.1% was used as negative control. Experiments were conducted in a growth chamber in the dark, at 25 ± 2°C. The evaluation of germination was performed 24 h after the introduction of seeds. The criterion for germination reading was the rootlet protrusion. The percentages of germination inhibition were calculated by comparison with the untreated control, using the following calculation: % inhibition = (*C* − *X*)/*C* × 100, where *C* is the number of seeds germinated in control and *X* is the number of seeds germinated in the test sample.

#### 2.3.2. Hypocotyls and Rootlets Growth

Seedling growth was evaluated five days after the introduction of the seeds with opening of plates. The percentages of inhibition were calculated by comparison with the untreated control, using the following formula: % inhibition = (*C* − *X*)/*C* × 100, where *C* is the average size of hypocotyl/rootlet in control and *X* is the average size of the hypocotyl/rootlet in the test sample [9, 10].

### 2.4. Statistical Analysis

Statistical evaluation of experimental data was performed by analysis of variance (ANOVA) and Tukey's comparison test, with the aid of the Graphpad Prism 5®. The value of *p* ≤ 0.05 was considered significant, and a confidence interval of 95% was adopted.

## 3. Results

### 3.1. Evaluation of Crude Ethanolic Extracts

The effect of crude extracts from cultivar BR-1 (aerial part, root, seed, and seed coat) was evaluated on the germination and growth of hypocotyls and rootlets of* L*.* sativa*. Seed and seed coat extracts showed highest percentages of inhibition of germination (47.78%), when compared to root (35.46%) and aerial part extracts (24.44%) (Table 1).

**Figure 1:**
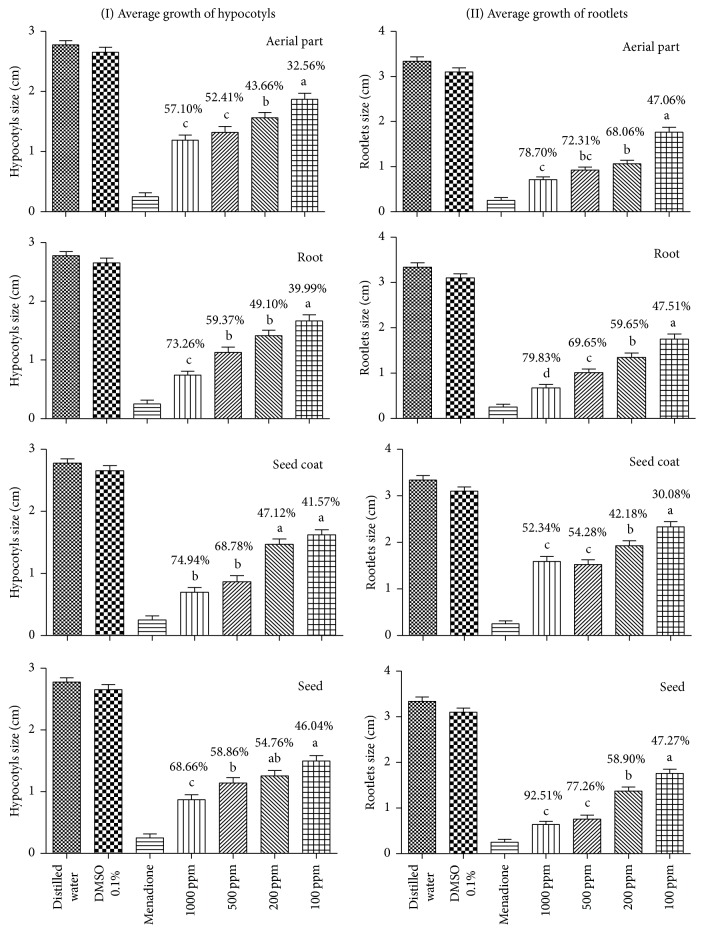
Evaluation of allelopathic activity of ethanolic extracts of* Arachis hypogaea* (cultivar BR-1) on the growth of hypocotyls and rootlets of* L*.* sativa* based on the average lengths of each organ and the inhibition percentages relative to distilled water control. The same letter means no statistically significant difference between the samples in each graphic, according to Tukey's test.

On the other hand, aerial part extract caused the highest percentages of growth inhibition in rootlets, when compared with hypocotyls. Similarly, root and seed extracts reached the higher percentage of inhibition on rootlets growth. Nevertheless, the percentage of inhibition induced by root extracts on the hypocotyls was higher when compared with the inhibition showed by extracts of aerial parts. Unlike these materials, seed coat extracts promoted greater inhibition on hypocotyls, when compared with rootlets, while the inhibition on rootlets was similar to those of the other extracts tested (Figure 1).

The IC_50_ (concentration of extract required to inhibit 50% of* Lactuca sativa* growth) was used for comparison of the activity of the different extracts from cultivar BR-1. Seed extracts showed the lowest IC_50_ values (72.98 ppm for hypocotyl and 77.43 ppm for rootlet development), while the root extract showed the highest values (367.5 ppm for hypocotyl and 472.5 for rootlet). The aerial part and seed coat extracts displayed similar values (91.46 ppm to 106.1 ppm) (Table 2).

The concentration corresponding to the lower IC_50_ values, obtained with cultivar BR-1 in order to inhibit the development of hypocotyls or rootlets, was used for the comparative analysis of extracts from other cultivars. The highest inhibition percentages (52.87% for hypocotyl and 58.25% for rootlet) were obtained with extracts from the aerial part of cultivar IAC 886 (Table 3).

### 3.2. Evaluation of Partitioned Extracts

The allelopathic activity of liquid-liquid partitions of the ethanolic extract of seeds of BR-1 (*n*-hexane, dichloromethane, ethyl acetate,* n*-butanol, and aqueous residue) was also evaluated. The inhibition caused by the nonpolar partitions, dichloromethanic (68.89%) and hexanic (57.78%), was much higher than that attained with the other partitions tested (13.33 to 16.67%) (Table 4).

**Figure 2:**
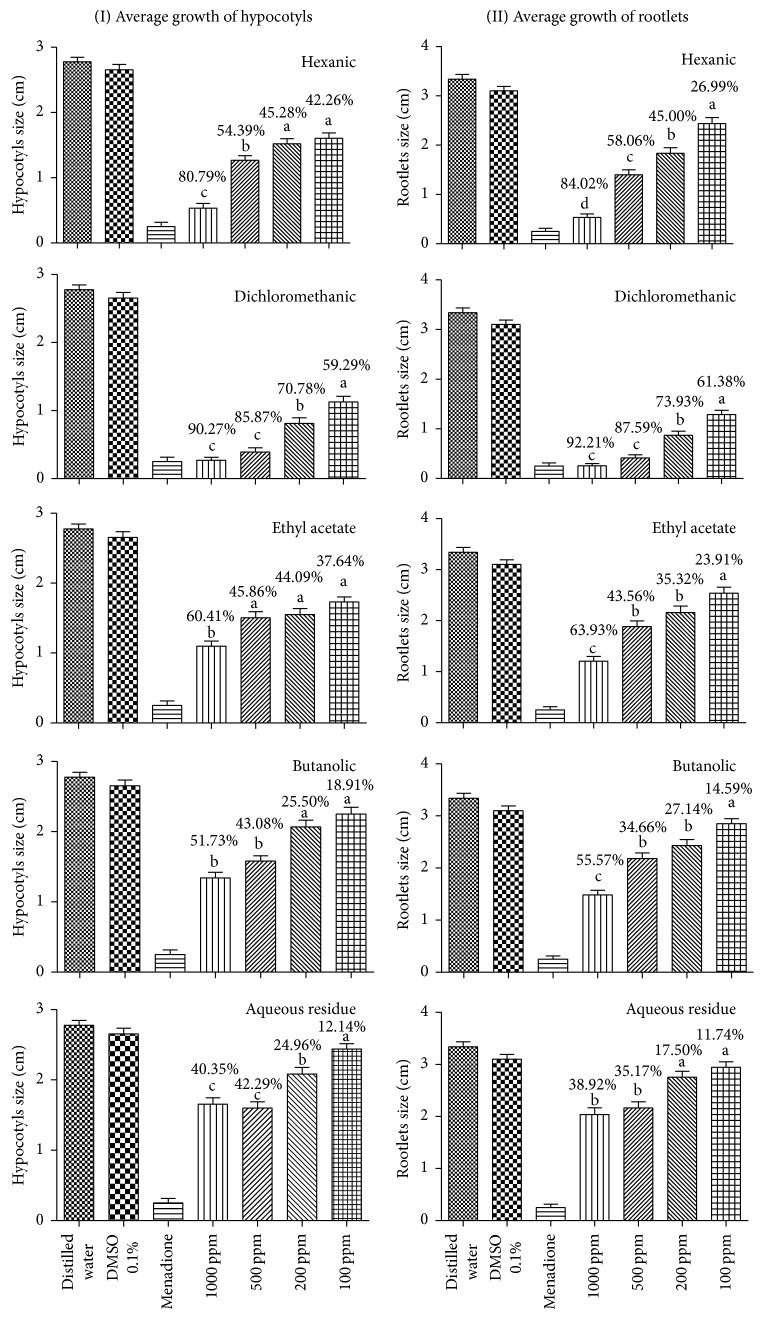
Evaluation of allelopathic activity of partitions of seed extracts of* Arachis hypogaea* (cultivar BR-1) on the growth of hypocotyls and rootlets of* L*.* sativa* based on the average lengths of each partition and the inhibition percentages are relative to distilled water control. The same letter means no statistically significant difference between the samples in each graphic, according to Tukey's test.

The inhibition percentages of growth of rootlets and hypocotyls varied according to the partition. The values obtained with the hexanic partition (80.79% for hypocotyl and 84.02% for rootlets at 1000 ppm) were higher than those achieved with the crude extract, which showed an inhibition of 68.66% on the growth of hypocotyls. Percentages found for rootlets were lower than those showed by the crude extract (until 92.51% at 1000 ppm). The dichloromethanic partition also caused the highest percentages of inhibition of growth of hypocotyls and rootlets (90.27 and 92.21, respectively, at 1000 ppm). Growth inhibition rates of ethyl acetate partition reached similar percentages when compared to crude extract. Butanolic partition and aqueous residue had the lowest percentages of inhibition, both in relation to other partitions and as compared to crude extracts (Figure 2).

As expected, the lowest value for IC_50_ was found with dichloromethanic partition on rootlets (19.70 ppm), while the highest value was found for aqueous residue (1360.0 ppm). All partitions evaluated, except dichloromethanic, showed lower IC_50_ values for hypocotyls as compared to rootlets growth (Table 5).

The dichloromethanic partition at IC_50_ (19.70 ppm) was evaluated for allelopathic activity on two weed species that affect* Arachis hypogaea* crops,* Commelina benghalensis* and* Ipomoea nil*. Although the inhibition of seed growth on* I*.* nil* was greater than on* C*.* benghalensis*, the inhibition of both species was lower than the observed in* L*.* sativa* (Table 6).

## 4. Discussion

In the present study, evaluations of allelopathic activity were made with ethanolic extracts of different materials as well as with liquid-liquid partitions of seed extracts from five cultivars of* A*.* hypogaea*. The allelopathic activity of peanut extracts has also been previously studied by other workers. Similar results to those obtained here were reported when the activity of aqueous extracts of different organs (root, stem, leaf, and flower) on the inhibition of germination of cucumber seeds was analyzed [11]. However, while these authors found highest allelopathic activity in root extracts, we attained best effects with ethanolic seed extracts. This difference shows that allelopathic activity of different organs of the same plant can vary according to the method used and the target species.

Inhibition of germination is one of the most parameters used to evaluate allelopathic activity. In this work, crude ethanol extracts of seeds and seed coats caused almost 50% of inhibition of germination of* L*.* sativa* seeds. In contrast, extracts of aerial parts of* Equisetum giganteum* and* Nephrolepis exaltata* had no effect on* L*.* sativa* seeds [12]. Nonpolar partitions (*n*-hexanic and dichloromethanic) of seed extract of* A*.* hypogaea* showed the highest capacities of inhibition of germination of* L*.* sativa*. The same values were found for equivalent partitions of aqueous leaf extract of* Sapindus saponaria*, which could also inhibit the germination of* L*.* sativa* [13].

In addition to seed germination of* L*.* sativa*, extracts were evaluated in relation to their effect on seedling growth. Among the organs evaluated, seed extracts showed the lowest IC_50_ for inhibiting both hypocotyls (72.98 ppm) and rootlets (77.43 ppm) growth. These values were even lower than those reported by other authors for resveratrol (IC_50_ = 90 ppm), a constitutive compound of* Arachis* species, also in relation to inhibition of* L*.* sativa*'s growth [14].

The concentrations used to determine the plant growth inhibitory activity usually vary in a wide range of high values. Certainly, they are unreasonable when thinking in farming use in the field, considering the possibility of environmental damage and disadvantaging the desired ecological balance. For example, rootlets of* L*.* sativa* showed an average length of 5.78 cm when treated with* Cuscuta campestris* extracts at a concentration of 100 ppm. This value was almost three times higher than that observed with extracts of* A*.* hypogaea* seeds for the same species and at the a similar concentration (less than 2.00 cm) [15]. In the evaluation of coffee fruit extracts* (Coffea arabica)*, the concentration required for the growth inhibition of hypocotyls and rootlets of* L*.* sativa* to be numerically similar to that found in our work was tenfold higher (1000 ppm) [16]. In a study of allelopathic activity of* Terminalia catappa* fruit extracts biomonitored by* L*.* sativa*, it was demonstrated that the IC_50_ of the dichloromethanic partition was 4.5 times reduced as compared to crude ethanol extracts [17]. In a paper with subfractions of* Cleome arabica*, a concentration of 3.000 ppm was necessary to inhibit 50% of* L*.* sativa* growth [18]. This concentration was much higher than that used for inhibiting 50% of the same species with the dichloromethanic partition (19.70 ppm) in the present study. Thus, the IC_50_ values found for* A*.* hypogaea* (77.43 ppm and 19.70 ppm for the crude extract and the dichloromethanic partition, resp.) for inhibiting growth of rootlets provide indications of the quality of the material studied to obtain substances with potential herbicide activity.

The allelopathic activity of certain species is very important to diminish the spread of weeds on crops. Thus, some plant extracts can act as natural herbicides, bringing many benefits to crops. Evaluation of the dichloromethanic partition of ethanolic extract of* A*.* hypogaea* performed in the present study showed inhibition of growth of weed, similarly to the results reported with extracts of* Terminalia catappa* fruits [17].

## 5. Conclusions

In conclusion, the results showed that aerial part, root, seed, and seed coat extracts of* A*.* hypogaea* could inhibit the germination of seeds and the growth of hypocotyls and rootlets of* L*.* sativa*. The partitions of ethanolic seed extract in* n*-hexane, dichloromethane, ethyl acetate, butanol, and aqueous residue can also have a positive allelopathic activity. Dichloromethanic partition of ethanolic seed extract is showed to be an environmentally sustainable alternative herbicide for weed management in other large-scale crops.

## Figures and Tables

**Table 1:** Inhibition of *L*. *sativa* seeds germination by crude ethanolic extracts of aerial part, root, seed coat, and seed of BR-1 cultivar from *Arachis hypogaea*.

Crude extract (BR-1—1000 ppm)	Inhibition of germination (%)
Aerial part	24.44^c^
Root	35.46^b^
Seed coat	47.78^a^
Seed	47.78^a^

The same letter means no statistically significant difference between the samples.

**Table 2:** IC_50_ of crude ethanolic extracts of cultivar BR-1 of *A*. *hypogaea* on the inhibition of *L*. *sativa* growth of hypocotyls and rootlets.

Crude ethanolic extract	IC_50_ (ppm)	
	Hypocotyl	Rootlet
Aerial part	99.91	96.68
Root	367.5	472.5
Seed coat	106.1	91.46
Seed	72.98	77.43

**Table 3:** Inhibition of *L*. *sativa* growth of hypocotyls and rootlets by crude ethanolic extracts of aerial part, root, seed coat, and seed of IAC 886, IAC Caiapó, IAC Tatu ST, IAC 8112, and BR-1 cultivars from *Arachis hypogaea*.

Cultivar	Inhibition in relation to control (%)							
	Crude extracts							
	Aerial part (96.68 ppm)		Root (367.5 ppm)		Seed coat (91.46 ppm)		Seed (72.98 ppm)	
	Hypoc.	Root.	Hypoc.	Root.	Hypoc.	Root.	Hypoc.	Root.
IAC 886	52.87^a^	58.25^a^	36.14^bc^	29.07^b^	29.75^b^	38.52^bc^	41.28^b^	40.15^ab^
IAC Caiapó	32.18^c^	35.27^d^	43.65^ab^	33.89^ab^	27.52^b^	32.97^c^	27.98^c^	26.78^c^
IAC Tatu ST	40.19^b^	38.59^cd^	32.13^c^	29.81^b^	30.18^b^	40.10^b^	39.58^b^	33.98^bc^
IAC 8112	42.03^b^	43.87^bc^	35.62^c^	28.32^b^	41.78^a^	45.21^ab^	35.52^bc^	36.27^b^
BR-1	46.81^ab^	50.00^ab^	50.00^a^	37.24^a^	44.09^a^	50.00^a^	50.00^a^	48.51^a^

The same letter means no statistically significant difference between the samples in each column.

Hypoc. = hypocotyl; Root. = Rootlet.

**Table 4:** Inhibition of *Lactuca sativa* seeds germination by partitions of seed extracts of BR-1 cultivar from *Arachis hypogaea*, at 1000 ppm.

Partitions (1000 ppm)	Inhibition of germination (%)
Hexanic	57.78^a^
Dichloromethanic	68.89^a^
Ethyl acetate	16.67^b^
Butanolic	16.67^b^
Aqueous residue	13.33^b^

The same letter means no statistically significant difference between the samples.

**Table 5:** IC_50_ of partitions of seed ethanolic extract of cultivar BR-1 of *A*.* hypogaea* on the inhibition of *L*. *sativa* growth of hypocotyls and rootlets.

Seed extract partitions of cultivar BR-1 *(Arachis hypogaea)*	IC_50_ (ppm)	
	Hypocotyls	Rootlets
Hexanic	93.93	216.8
Dichloromethanic	23.88	19.70
Ethyl acetate	42.95	447.6
Butanolic	361.6	976.6
Aqueous residue	310.7	1360.0

**Table 6:** Inhibition of weed plants (*Commelina benghalensis *and *Ipomoea nil*) growth of hypocotyls and rootlets by dichloromethanic partition of seed extract of cultivar BR-1 of *Arachis hypogaea*.

Species	Inhibition of seed germination of weed plants by dichloromethanic partition (IC_50_ = 19.70 ppm) (%)	
	Hypocotyl	Rootlet
*Commelina benghalensis*	14.29^b^	23.56^b^
*Ipomoea nil*	33.78^a^	38.11^a^

The same letter means no statistically significant difference between the samples.
